# OUT OF THE MEDICINE CABINET: NONPHARMACOLOGICAL APPROACHES TO IMPROVE COGNITION AND ASSOCIATED FUNCTIONS

**DOI:** 10.1093/geroni/igad104.1202

**Published:** 2023-12-21

**Authors:** Ashley Curtis, Constance Visovsky

## Abstract

There is a lack of pharmacological agents for improving cognition, and the negative impact of polypharmacy on cognitive and physiological functioning in aging adults is well established. This has prompted investigation of non-pharmacological interventions aimed at improving cognition and associated functions. This symposium will provide a comprehensive evaluation of several of these promising evidence-based behavioral approaches. Paper 1 will describe several computerized cognitive training programs for improving cognition and sleep in middle-aged and older adults. Paper 2 will evaluate short-term complex music training versus music listening on cognitive function in older adults. Paper 3 will describe the impact of cognitive behavioral and virtual reality treatments for insomnia on sleep, cognition, arousal, and mood in several primarily middle-aged and older adult populations with chronic insomnia. Paper 4 will examine physical differences in gait function of older adult breast cancer survivors, providing insight into fall risk indicators, and targets for behavioral intervention. Paper 5 will describe results from a randomized clinical trial evaluating a mindfulness-based stress reduction for improving self-reported and objective cognition as well as fatigue. As Discussant, Dr. Constance Visovsky will integrate research findings and discuss areas for future research investigation. In sum, this symposium will provide unique insight into the impact of alternative approaches ‘beyond the medicine cabinet’ for improving not only cognition, but other areas of associated functioning in mid-to-late life. Thus, this session will help to inform interventions that can be delivered online and in the community to help mitigate risk and/or delay onset of cognitive decline.

